# Lions as Bone Accumulators? Paleontological and Ecological Implications of a Modern Bone Assemblage from Olduvai Gorge

**DOI:** 10.1371/journal.pone.0153797

**Published:** 2016-05-04

**Authors:** Mari Carmen Arriaza, Manuel Domínguez-Rodrigo, José Yravedra, Enrique Baquedano

**Affiliations:** 1 Departamento de Geología, Geografía y Medio Ambiente, Universidad de Alcalá, Edificio de Ciencias. Campus Externo. Ctra. A-II-km 33,600 C. P. 28871 Alcalá de Henares (Madrid), Spain; 2 Instituto de Evolución en África (IDEA), Museo de los Orígenes, Plaza de San Andrés 2, 28005, Madrid, Spain; 3 Departamento de Prehistoria, Universidad Complutense, Prof. Aranguren s/n, 28040 Madrid, Spain; 4 Museo Arqueológico Regional de la Comunidad de Madrid, Plaza de las Bernardas s/n, 28801, Alcalá de Henares (Madrid), Spain; University of Oxford, UNITED KINGDOM

## Abstract

Analytic models have been developed to reconstruct early hominin behaviour, especially their subsistence patterns, revealed mainly through taphonomic analyses of archaeofaunal assemblages. Taphonomic research is used to discern which agents (carnivores, humans or both) generate the bone assemblages recovered at archaeological sites. Taphonomic frameworks developed during the last decades show that the only large-sized carnivores in African biomes able to create bone assemblages are leopards and hyenas. A carnivore-made bone assemblage located in the short-grassland ecological unit of the Serengeti (within Olduvai Gorge) was studied. Taphonomic analyses of this assemblage including skeletal part representation, bone density, breakage patterns and anatomical distribution of tooth marks, along with an ecological approach to the prey selection made by large carnivores of the Serengeti, were carried out. The results show that this bone assemblage may be the first lion-accumulated assemblage documented, although other carnivores (namely spotted hyenas) may have also intervened through postdepositional ravaging. This first faunal assemblage potentially created by lions constitutes a new framework for neotaphonomic studies. Since lions may accumulate carcasses under exceptional circumstances, such as those documented at the site reported here, this finding may have important consequences for interpretations of early archaeological and paleontological sites, which provide key information about human evolution.

## Introduction

The lion (*Panthera leo* Linnaeus, 1758) is the largest carnivore of Africa and one of the best studied mammal species in the Serengeti National Park, as well as in other African ecosystems [1–3]. The genus *Panthera* first appears about 3.85–3.63 million years ago at Laetoli in Tanzania [4]. During the Pleistocene, large lion-like felids were distributed across most of Africa, Europe, Asia, North America and the northern part of South America [5]. Nowadays, lions have undergone dramatic range retraction but they remain widespread in Africa, from the southern Sahara to South Africa [6]. However, lions have not played a significant role in the interpretation of Plio-Pleistocene assemblages.

During the last few decades, archaeologists have developed methodological approaches to test hypotheses of hominin behaviour. One of the main disciplines dealing with this goal is taphonomy. This discipline focuses on discriminating the agency in the accumulation of prey bone assemblages at archaeological sites. Classical approaches and methods, such as the study of skeletal representation, bone breakage patterns, age class profiles and bone surface modification (e.g., cut marks or tooth marks), were developed within the framework of actualistic studies [7–14]. These analyses reveal which bone accumulations are due to natural deaths, and, more importantly, which are due to carnivores or hominins (or the interaction of both). Taphonomic research enables the testing of hypotheses regarding the strategy of carcass acquisition by humans, interspecific competition with other carnivores, and hominin social behavioural patterns. This is of great importance in human evolution studies, where relevant paleoanthropological sites such as those in Olduvai Gorge (Tanzania) have been interpreted as hominin-carnivore palimpsests, and are still under discussion [15–38].

The accumulation of skeletal remains by non-human predators over time can produce bone deposits. For instance, birds of prey, corvids and gulls can produce bone accumulations through their pellets [39–41] and small carnivores through their scats [42]. Large carnivores produce bone accumulations when prey is moved rather than consumed at the kill site. The consumption locus is a function of the trophic level, habitat (ecological and physical conditions) and the seasonal availability of resources [43]. So far, actualistic studies on macromammalian carnivores have documented bone-accumulating capabilities in dens for the following taxa: spotted hyena (*Crocuta crocuta* Erxleben, 1777) [44–45]; stripped hyena (*Hyaena hyaena* Linnaeus, 1758) [46–48]; brown hyena (*Hyaena brunnea* Thunberg, 1820) [49–50]; and leopard (*Panthera pardus* Linnaeus, 1758) [8,48,51].

Leopards and hyenas are thought to be the only carnivores that frequently transport their prey in savannah ecosystems [8,43–44]. There are clear differences between hyaenid and felid taphonomic characteristics. Felids, for instance, transport a larger number of complete carcasses, so that the axial skeleton (e.g., ribs, vertebrae, scapulae and coxae) and compact limb bones (e.g., phalanges, carpals/tarsals) are better represented, and bone surfaces display fewer tooth marks than carcasses ravaged by hyaenas [8,29,37,52]. It has been argued that the prey-age patterns also differ: felid ambush strategies usually produce catastrophic age profiles, in contrast to the attritional age profiles of cursorial hyaena predation [53–54]. In the case of hyaenas, Stiner [55] and Cruz-Uribe [56] described some criteria to distinguish the bone accumulations made by these carnivores, such as the higher relative abundance of both antlers and horns, the low relative abundance of compact bones (phalanges, carpals/tarsals, sesamoids), different skeletal representation depending on prey size, and relative abundance of carnivore remains, among others. Further research has refuted some of these criteria and has shown the great variability of hyaenas as taphonomic agents [57–59]. In spite of the prolific information on hyaena behaviour, further work is being undertaken to understand the variability of the taphonomic signatures among hyenid species in different ecological contexts, in order to create accurate analogs to compare with the bone assemblages recovered in archaeological sites [59,60–63].

In contrast to hyenas and leopards, lions consume their prey at the kill site, as it is a gregarious top predator and the ecosystem’s largest extant carnivore [43]. Because lions do not systematically transport prey, there is, ostensibly, little possibility for them to create bone assemblages. One interpretation of early archaeological sites is that carcasses abandoned by felids were habitually transported to certain locations by early hominins for scavenging purposes [18]. This is the “passive scavenging” hypothesis, in which medium and large ungulate carcasses abandoned by large felids in riverine alluvial forests during the dry season offered scavenging opportunities to early hominins [15]. Alternative hypotheses have produced the ‘hunting versus scavenging’ debate, which has resulted in different interpretations of early hominin behaviour in Bed I at Olduvai Gorge, not least the FLK Zinj site (cf. ‘Summary’ of the controversy in [29]).

Previous interpretations of early archaeological sites assumed that large felids such as lions do not accumulate carcasses. Thus, the presence at early Pleistocene sites of skeletons of medium-size ungulates bigger than those normally hunted by the leopard could not be attributed theoretically to a felid. However, in some cases, large felids have been proposed as the primary accumulating agent at some paleoanthropological sites [29,64], but, problematically, these interpretations lacked a modern proxy.

The present study analyzes an extensive collection of modern wildebeest carcasses seasonally accumulated by the same type of carnivore in the Southern Serengeti. This carnivore site is located in Olduvai Gorge (Tanzania), which lies in the short-grassland ecological unit of the Serengeti National Park (SNP). The accumulating agent in the site presented here is known to be a carnivore because bones with conspicuous damage inflicted by a carnivores were collected in 2012, and new bones were found the following year at the same site. This implies that an accumulating agent has been actively bringing carcasses into the site between 2012 and 2013. Furthermore, the age-class profile documented at the site does not correspond with a catastrophic event [65]. The only prey present at the site is the blue wildebeest (*Connochaetes taurinus* Burchell, 1823), making the Olduvai carnivore site (OCS) the first documented faunal assemblage in Africa containing the remains of just one ungulate species. The aim of this paper is to identify the carnivore(s) involved in the accumulation and modification of these wildebeest carcasses. Analyses of skeletal-part representation, bone density, breakage patterns and tooth marks, along with an ecological approach to prey selection by the large carnivores of the Serengeti were carried out. The results show that this bone assemblage may be the first made by lions ever documented, although other carnivores (namely spotted hyena) may have acted in the modification of the bone assemblage post-depositionally. Because we now know that lions may accumulate carcasses, this finding requires a re-evaluation of the interpretations of some early archaeological sites. Furthermore, the new data presented here may be useful in future taphonomic research carried out in Pleistocene sites from Africa, Europe, Asia, North America and the northern part of South America, where large lion-like felids may have generated bone assemblages in those paleoecosystems [5].

## Material and Methods

All necessary permits were obtained for the described study (Tanzania Commission for Science and Technology: COSTECH permit: 2014-174-ER-2006-115). The specimen numbers studied were 1–4533. The specimens involved in the study are publicly deposited in the Instituto de Evolución en África (IDEA, Madrid, Spain) and in the Research station of Olduvai (Olduvai Gorge, Ngorongoro Conservation Area, Arusha, Tanzania).

**Fig 1:**
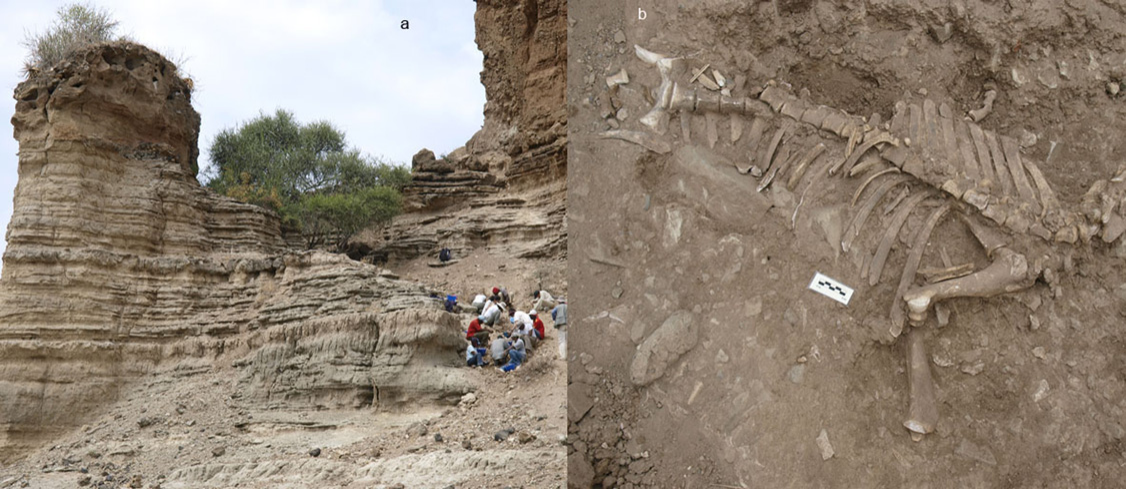
Location of the OCS site. a) Excavation work on the slope where the wildebeest bones were concentrated in 2012. b) One of the wildebeest carcasses recovered in 2012.

Olduvai Gorge is a valley at the western margin of the Eastern Rift Valley in northern Tanzania. The valley cuts the Serengeti Plain, which extends about 110 km northeast toward Lake Victoria [66]. Olduvai Gorge is located in the short grassland ecological unit of the Serengeti ecosystem [67]. The Olduvai Paleoanthropology and Paleoecology Project (TOPPP) has been conducting on-going research at Olduvai Gorge since 2006. During the 2012 field season (carried out in June and July), the team located a modern carnivore-made bone assemblage in a gully close to the third fault of the gorge. It is composed exclusively of wildebeest bones with conspicuous carnivore marks. The bones were concentrated on a slope situated close to a river course with an associated riparian forest (Fig 1). During the 2013 field season, new fresh wildebeest bones were found at the site, and we concluded that the site was active.

In 2012, the team collected and excavated all the bones found at the slope of the site, along with bones dispersed at a distance up to a radius of 15 meters. In total, 4107 wildebeest bones were recovered. In 2013 the team collected all the bones found at the site: 426 wildebeest bones. This amounts to a sample of 4533 wildebeest bones in total.

### Skeletal part representation and bone density

In order to determine how many individuals were present and their anatomical profiles, we quantified skeletal part representation. Each specimen was identified according to element, bone portion (in the case of limbs, these are proximal/distal epiphysis or diaphysis) and bone section (in the case of limbs, proximal/distal epiphysis, near-epiphysis shaft, and midshaft). The minimum number of elements (MNE) was calculated following Yravedra and Domínguez-Rodrigo [68]. This comprehensive method takes into account anatomical landmarks, the location of the shaft fragment on the complete bone as well as the size of individuals and relative age as reflected in cortical texture and body size. Minimal numbers of individuals (MNI) were obtained by siding paired bones and considering their age [69]. Several indices were derived from the MNE estimates: a fragmentation index (NISP:MNE ratio) and the minimum number of animals units (MAU) [7]. In order to obtain a standardized percentage (% MAU) these values were divided by the highest MAU value in the assemblage. The %MAU values were correlated with density estimates for the wildebeest skeleton, in order to assess density-mediated biasing processes [70]. We averaged density values for several scan sites to achieve the most conservative estimate for each complete bone element.

In order to determine the carnivore type involved in the accumulation of the carcasses, the skeletal representation documented in three spotted hyaena dens -Syokimau [60], Amboseli [48] and Masai Mara [71]- and 3 leopard dens -Hakos River and Portsmut [8] and the John Nash Nature Reserve den [51]- were compared with the results obtained from the OCS assemblage through Principal Component Analysis (PCA), cluster analysis (CA) and Random Forest (RF) performed in R (www.r-project.org). Both PCA and CA were made using the "FactoMineR" library.

Blue wildebeest is size 3 as defined by Bunn [10]. The skeletal part representation data taken from the modern spotted hyena den samples are those which correspond with prey size 3 (Syokimau- MNE = 177; Masai Mara- MNE = 666; Amboseli- MNE = 108). The skeletal part representation taken from the modern leopard dens studied by Brain [8] are those corresponding also to prey size 3 and prey size 2–3 (Portsmut- MNE = 15; Hakos River- MNE = 128). The skeletal part representation from the modern leopard den studied by Ruiter and Berger [51] corresponds to prey size 2, because they did not recover any prey species from size 3 (MNE = 221).

Anatomical profile analysis was carried out *via* PCA, CA and RF because these statistical methods are powerful exploratory/confirmatory multivariate methods (PCA, CA) and also because one of them is constructed around a great classifying algorithm (RF). PCA distributes data in a Euclidean space by using maximizing variance differences. It reduces multivariate dimensionality using components (structured as combinations of variables using eigenvalues). CA groups sets of objects according to their overall similarity or/and differences depending on the method. In the present analysis, the unweighted pair-group average (UPGA) linkage method was used. CA uses (in the present work) Euclidean distances and, therefore, makes it quite compatible with PCA, with which it can even be combined (by doing CA on PCA loading scores).

Regression methods built in the form of a tree, which create decision nodes indicating a consecutive chain of variables with their corresponding attributes. This is done in the form of branches showing the decision´s choices, which end up in terminal nodes displaying the result of classification according to specific combinations of decisions. This decision process is carried out through recursive partitioning of data. One of the major advantages of decision trees is that they accept combinations of numerical and categorical variables and is not limited by requirements of typical parametric tests, such as normality or heterocedasciticy. RF averages ensembles of decision trees. The algorithm uses a small random number of the data set variables, instead of all the variables. Each selection produces an independent tree. Bootstrap aggregation, more commonly known as bagging, is the common procedure of random forests, which splits a training data set into multiple data sets derived from bootstrapping. RF thus produces a selection of variables that are important for correct classification of the analytical set. RF produces hundreds of trees that are repeatedly fitted to bootstrapped sets of data. The results are contrasted against a validation test, from the observations (about one third) not used for the training data set. These observations are referred to as out-of-bag (OOB) observations. RF produce estimates on how many iterations are needed to minimize the OOB error. The importance of each response variable is determined by mean decreased error (MDE) for regression trees (RT), whereas the Gini index is more useful for classification trees (CT). After selecting a number of tress (e.g., 2000) the algorithm averages the results and produces a robust classification method, which avoids overfitting of results to data, as is more common in standard decision and regression trees. For the present study, the “randomForest” R library was used.

For the analysis of skeletal part profiles, the variables used in the PCA, CA and RF were %MAU of each skeletal element: skull, mandible, vertebrae (cervical, thoracic, lumbar and caudal), ribs, scapulae, pelvis, humerus, radius, ulna, femur, tibia, metapodials, carpals/tarsals, phalanges and others. Thus, a total of 19 variables were used. The analysis of complete long bones involved the six long bones (humerus, radius-ulna, metacarpal, femur, tibia and metatarsal). The variables used for the analysis of furrowing on long bones involved the proximal and distal ends of all long bones but metapodials, which are commonly abandoned unmodified by felids. This implied the use of 9 variables (proximal and distal end of humerus, radius, femur, tibia and proximal ulna). A more thorough description of each of the variable sets and their results can be seen in S1 File.

### Orientation patterns

The orientation pattern of bones indicates the degree of post-depositional disturbance in assemblages. Skeletal part representation may be biased due to physical processes (such as water flow) or gravity-induced resedimentation occurring during the biostratinomic stage of site formation [72]. These processes can be detected through the analysis of bone orientations. The orientation of a random sample of 239 bones from the OCS bone assemblage was measured following criteria summarized in Domínguez-Rodrigo and García-Pérez [72]. Three statistical analyses were carried out to test the isotropy of the sample: Rayleigh, Kuiper and Watson tests performed in R.

### Breakage patterns

The frequency of complete long bones was calculated and compared with the frequencies reported from the Amboseli and Masai Mara spotted hyaena dens [48] for prey size 3. The percentage of circumference preserved in each limb bone was recorded following the system created by Bunn [10]: type 1 shafts preserve < 50% of their circumference, type 2 shafts preserve more than 50% of their circumference but less than 100% and type 3 shafts preserve 100% of their circumference. These results were compared to those recorded from the Syokimau spotted hyaena den for prey size 3 [60]. Additionally, the fracture type (green or dry) was noted for each fragment [73] and the angles of the oblique fractures (angle formed by the fracture surface and the bone cortical surface) were recorded using a goniometer [74–75]. The results were compared with those reported in experiments carried out with carnivores (static loading) and hammerstone percussion (dynamic loading) [74].

Notches along the breakage planes were recorded and assigned to one of the following types: single (A), incomplete (B), double-overlapping (C) and double-opposing (D) [76].

### Bone surface modification

Following the criteria summarized by Blumenschine et al. [77], all conspicuous and inconspicuous tooth marks on bone specimens were identified with the aid of a 15 x hand lens. The sample was stratified according to bone density: cancellous bone (from epiphyseal sections) and dense cortical bone (from diaphyseal sections). Conspicuous pits were measured in length and breadth, following the methodology described in Andres et al. [78]. The results were compared to those recorded for lion and spotted hyaena [78], and leopard and cheetah [30].

Carnivores show a redundant carcass consumption behavior, which leaves a regular pattern in the way that long bones are modified [7]. Furrowing patterns were recorded from the OCS sample and then were compared with the patterns reported for lion (data from [79], and unpublished data from Gidna) and spotted hyaena kills [80] for prey size 3. A recent study has developed a new methodology to identify bone destruction made by different kinds of carnivores [81]. Domínguez-Rodrigo et al. [81] devised the term “taphotype” to define each bone modification type according to damage documented per long bone quadrant. The taphotypes documented from lion and spotted hyaena kills show clear differences on long bone modification patterns [81]. We applied this new methodology to the OCS sample, and the results were then compared with the taphotypes recorded from lion and spotted hyaena kills, through a CA (correspondence Analysis) performed in R.

## Results

### Skeletal part representation

**Fig 2:**
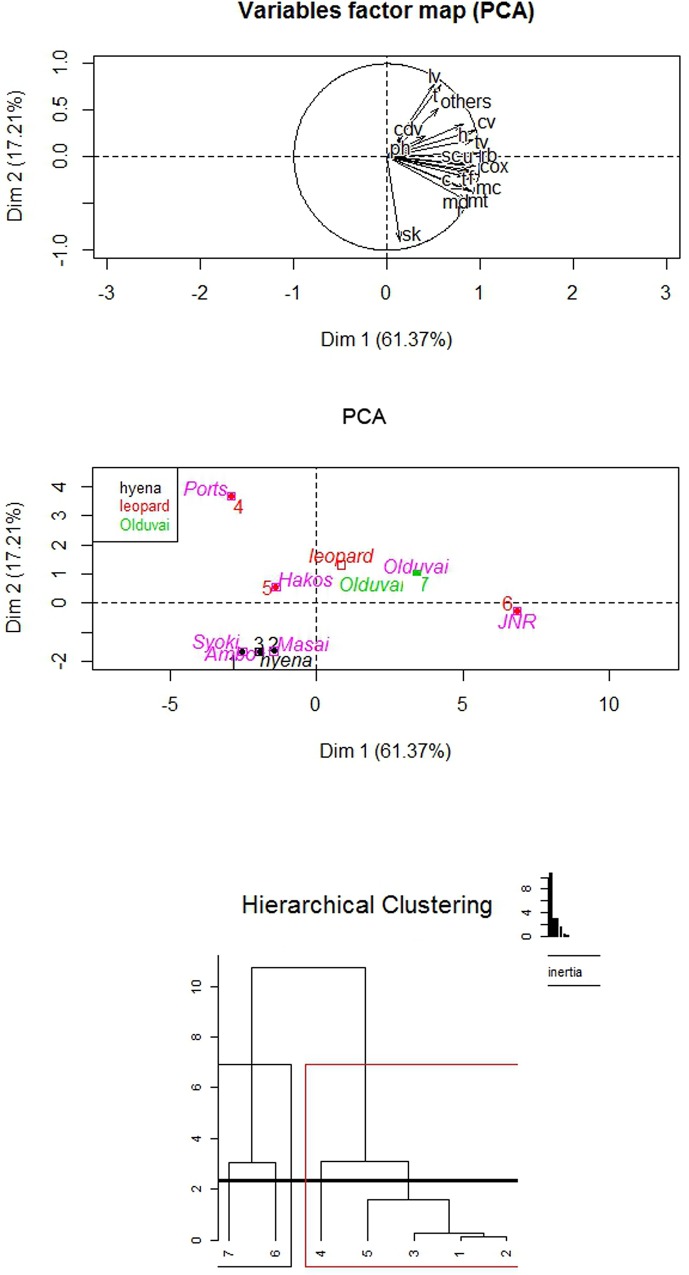
Variable factor map (a) and distribution of samples (b) in the two-component PCA solution for the analysis of skeletal part profiles and their use in discriminating felid and hyenid bone assemblages. C, Hierarchical cluster analysis using the same samples as PCA. Key for PCA: sk: skull, md: mandible, cv: cervical vertebrae, tv: thoracic vertebrae, lv: lumbar vertebrae, cdv: caudal vertebrae, rb: rib, sc: scapula, cox: innominate, h: humerus, r: radius, u: ulna, mc: metacarpal, f: femur, t: tibia, mt: metatarsal, c_t: carpal/tarsal, ph: phalanges. Key for CA: for leopard dens (numbers 4,5 and 6; data from ref 8, 51), spotted hyaena dens (numbers 1,2 and 3; data from ref 60, 48, 71) and for the OCS (number 7).

Fifty-five wildebeest were recovered at the site: 5 yearlings and 50 adults. Table A in S1 File shows the NISP, MNE, MAU, % MAU and NISP/MNE. Wildebeest are represented by fairly complete skeletons at the OCS (Fig 1). This could indicate a complete transport of the carcasses carried out by the carnivore, which is typical of felids. PCA and CA support this hypothesis. A PCA yielded a two-component solution that accounted for 78.58% of sample variance. The first component alone explained more than 60% of the total sample variance. This component was determined (with correlation scores >0.9) by the distribution of ribs, innominate, ulnae, cervical vertebrae, thoracic vertebrae, mandibles and metacarpals in all the assemblages. Lumbar vertebrae, skulls and tibiae were the most important variables for the second component (Table B in S1 File). As seen in Fig 2, skeletal part representation differs according to carnivore type. The Euclidean spatial distribution clearly separates the frequency of bone elements found at the hyaena dens from that found in leopard-made bone accumulations. The skeletal representation from OCS is similar to the skeletal patterns recorded at leopard assemblages.

**Fig 3:**
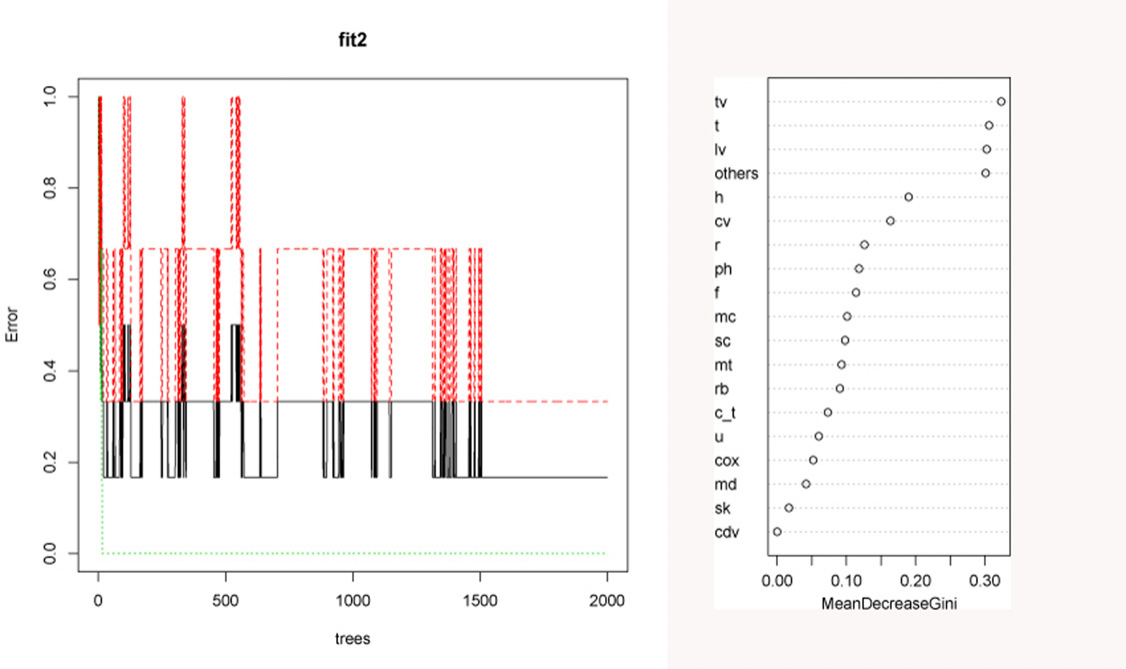
Random forest analysis for the skeletal part representation. Left, error rate for hyenas (Green), felids (Red) and combined oob error rate (black). Stabilization of error was achieved after 1500 decision tress. Right, gini index showing the most important variables for the most accurate classification. The most influential variables are shown on top. (data from ref 8, 51, for leopard dens and data from ref 60, 48, 71, for spotted hyaena dens). Key: cv: cervical vertebrae, tv: thoracic vertebrae, sk: skull, lv: lumbar vertebrae, ph: phalanges, cdv: caudal vertebrae, f: femur, rb: rib, c_t: carpal/tarsal, u: ulna, r: radio, h: humerus, cox: innominate, t: tibia, md: mandible, mt: metatarsal, mc: metacarpal, sc: scapula.

**Fig 4:**
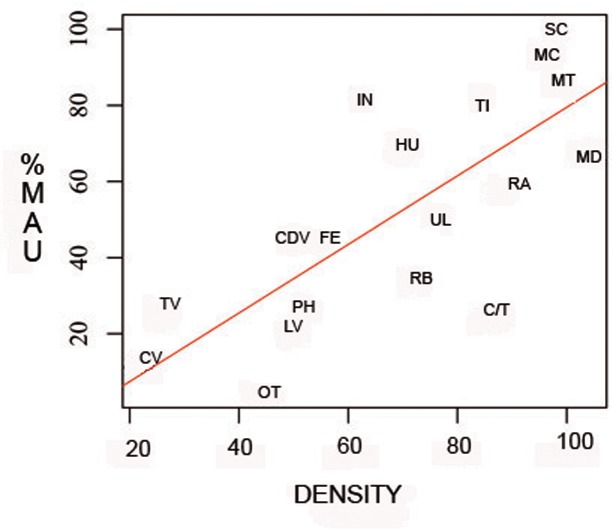
Plot showing survivorship values (%MAU) at OCS compared to density (%) values for the wildebeest skeleton (ref 70). Key: cv: cervical vertebrae, tv: thoracic vertebrae, ot: others, lv: lumbar vertebrae, ph: phalanges, cdv: caudal vertebrae, fe: femur, rb: rib, c/t: carpal/tarsal, ul: ulna, ra: radio, hu: humerus, in: innominate, ti: tibia, md: mandible, mt: metatarsal, mc: metacarpal, sc: scapula.

The RF test produced a solution in which the OOB error stabilized after 1500 trees (Fig 3). The result produced an OOB estimate of the error rate of 16.67%. The solution is correct over 83% of the time. The mean decreased Gini value showed that the most important variables were thoracic vertebra, tibia, lumbar vertebra and the category called “others” mostly composed of cancellous bones (sacrum, sternum, sesamoid, patella, malleolus). The probability yielded by the RF that the carcasses from the OCS were transported by a felid is around 76%, thus corroborating the results obtained in the PCA-CA. The study of the bone density shows that there is a significant relationship between survivorship of the bone elements and their density values (Spearman's rank correlation rho = 0.7565892; p-value = 0.0002791). This suggests that density-mediated factors and processes biased the original bone accumulation in a meaningful way (Fig 4). This implies that carcasses prior to the intervention of these density-mediated factors must have been even more complete than reported here.

### Orientation patterns

Table C in S1 File shows the results of the statistical analyses of isotropy applied to the OCS assemblage. The Rayleigh test value shows that the sample is anisotropic. The Kuiper and Watson tests show more than one recurrent orientation is documented in the sample. This indicates that some post-depositional disturbance, probably due to the combination of water and steep slope (during the rainy season) may have rearranged bones and even transported some of them downslope. This may have some repercussions to the differential distribution of all skeletal elements in the collection.

### Breakage patterns

**Fig 5:**
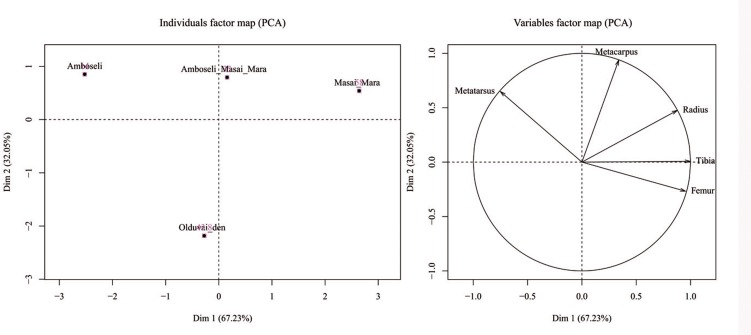
Variable factor map (right) and distribution of samples (left) in the two-component PCA solution analyzing complete long bones frequencies from Amboseli and Masai Mara spotted hyaena dens (data from ref 48) and OCS.

Table D in S1 File shows the frequency of complete long bones found at OCS. These data were compared to the frequency of complete long bones recovered from two spotted hyaena dens, Amboseli and Masai Mara (data from Kerbis-Peterhans, 1990). No data on this variable has been published for felid-accumulated assemblages. The PCA shows clear differences between the spotted hyaena dens and the OCS assemblage (Fig 5). The OCA yielded a two-component solution, which explains 99.28% of the total sample variance. The first dimension (67.23% of sample variance) is accounted by the frequency of complete tibiae, femora and humeri. The second dimension is accounted for the representation of complete metapodials (Table E in S1 File). This two-component solution suggests that OCS does not resemble any of the versions of the assemblages documented for spotted hyenas, because bones (especially metapodials) survive complete in much higher rates than documented among hyenas.

A total number of 1442 bone specimens could be identified as bearing green and/or dry breakage planes (Table F in S1 File). Of these, 593 specimens showed green breaks and 996 had dry breaks. Most of the dry breaks were observed on axial bones (n = 641), with the remainder documented on long bones (n = 351) and compact bones (n = 3). Green breaks were documented in higher numbers on long bones (n = 341) and axial bones (n = 248), and less frequently on compact bones (n = 3).

A total of 37 notches were identified. Most of the notches were either of type D (n = 12) or incomplete (n = 10) (Table G in S1 File).

Another diagnostic variable is platform angle. Static loading from carnivore breakage creates more right-angled fracture planes than hammerstone-broken bones through dynamic loading. Alcántara et al. [74] showed that oblique planes were more informative of the type of loading (dynamic versus static) than longitudinal and transversal planes. Table H in S1 File shows the results for the fracture angle for the OCS, which are similar to those obtained for oblique breakage planes after carnivore consumption.

**Fig 6:**
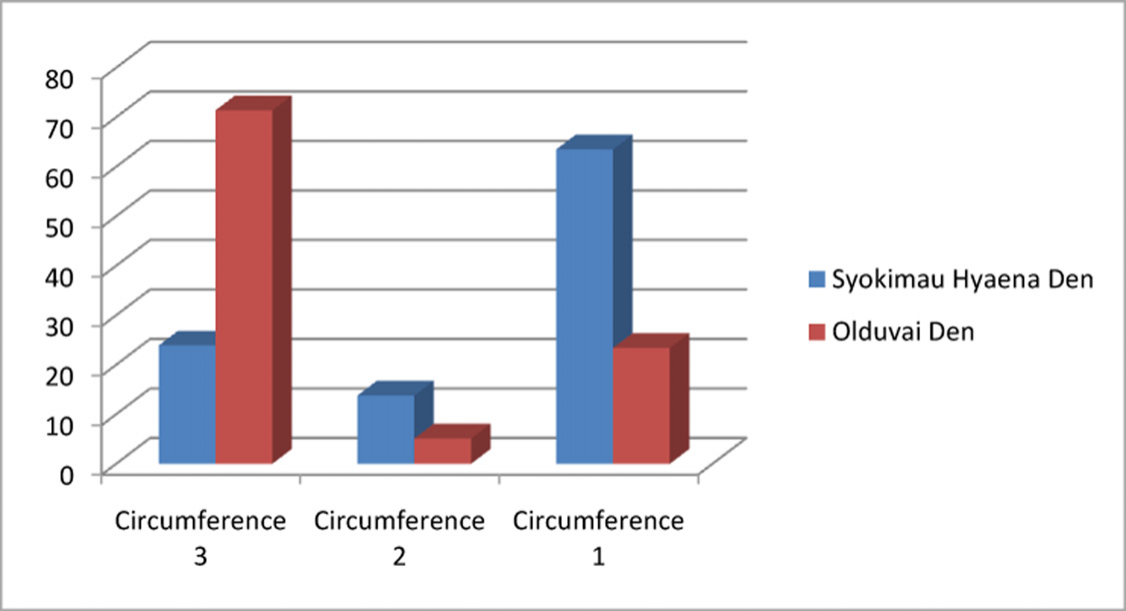
Limb shaft circumference type for Syokimau spotted hyaena den (data from ref 60) and OCS. See definition of types in text.

When analyzing the distribution of Bunn’s [10] circumference types on long bones, circumference type 3 (complete section) is the most frequent and type 1 and 2 are the least frequent at the OCS (Fig 6). This pattern is different from those found at the spotted hyaena dens where circumference type 1 is the most frequent, because these carnivores cause high degrees of bone breakage.

### Bone surface modification

About 4.5% of the OCS specimens bear tooth marks (Table I in S1 File). Cranial elements, compact bones and the axial skeleton (vertebrae/ribs) are the least tooth-marked elements. Although the axial skeleton was not heavily modified by carnivores, vertebrae show some carnivore damage (Table J in S1 File). The thoracic and lumbar vertebrae were the most affected axial elements. On the other hand, long bones are the most highly tooth-marked elements. Humerus and femur are the most tooth-marked long bones and tibia is the least tooth-marked. The midshaft portions of long bones are more frequently tooth-marked than epiphyses (Table K in S1 File).

**Fig 7:**
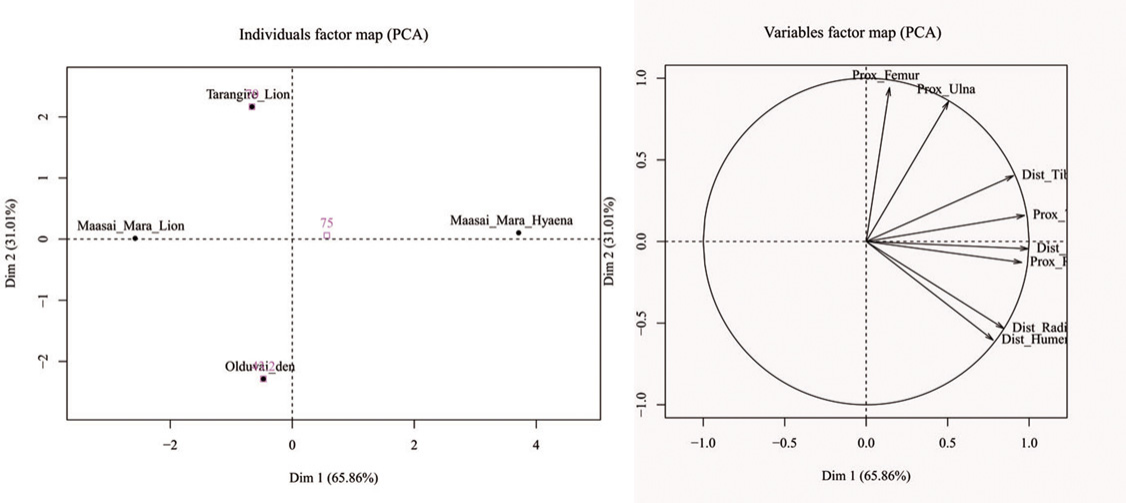
Variable factor map (right) and distribution of samples (left) in the two-component PCA solution analyzing furrowing patterns for lions (data from ref 79 and unpublished data from Gidna), spotted hyaenas (data from ref 80) and OCS. The OCS assemblage shows similar amount of furrowing to the lion samples (all with negative values in the first component), but more than the lions because distal ends of humeri and radii are more furrowed (as in hyenas), hence the separation on the second component

**Fig 8:**
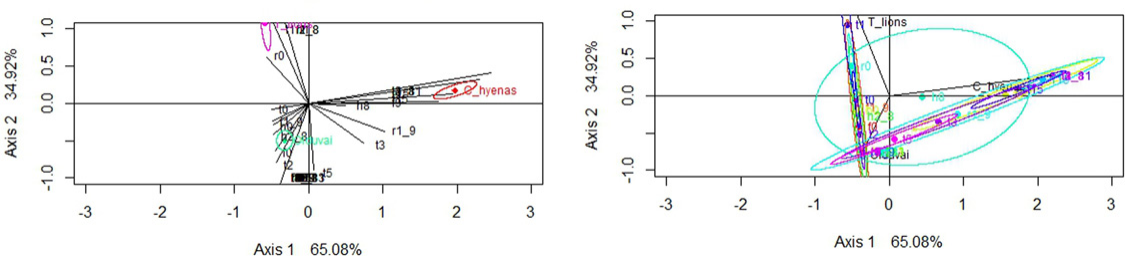
Taphotypes for lions (pink), spotted hyaena (red) (data from ref 81) and the OCS (green), according to CA. Notice the separation of the confidence interval between the felid and the hyenid samples and the overlap of both with the OCS sample, which is most closely associated with the confidence ellipse of the felid sample.

Fig 7 shows that the furrowing patterns reported at spotted hyaena dens and in lion kills are different. A PCA yielded a two-component solution, which explains 96.87% of the total sample variance. The first component accounts for 65.86% of the sample variance. The furrowing patterns that are most influential in the loading scores of the PCA are those found in the distal femur, proximal tibia and proximal radius (Table L in S1 File). The result documented in the OCS sample do not coincide with the patterns of these carnivores (Fig 7). OCS coincides with the Tarangire lion sample on the first component, but diverges from it in the second component, with a higher damage reported for distal humeri and distal radii, suggesting a two-patterned process (felid-hyenid?), instead of just a single felid or hyenid scenario. The taphotype study shows the same distribution (Fig 8). A correspondence analysis (CA) yielded a two-dimension solution with an ideal result, explaining 100% of the sample inertia. The first dimensions alone accounted for 65% of the sample inertia. The CA clearly separates the damage inflicted by the spotted hyaena and lion on long bones, but the OCS assemblage shows no direct match to any of these carnivores. Nevertheless, when the confidence intervals are introduced in the CA analysis (Fig 8), distinctive taphotypes of the lion and spotted hyaena are included in the OCS sample (see Figures B, C, D, E, F in S2 File for taphotypes examples). This is a clear indication that OCS is the result of the intervention of more than one agent in the modification of bones.

**Fig 9:**
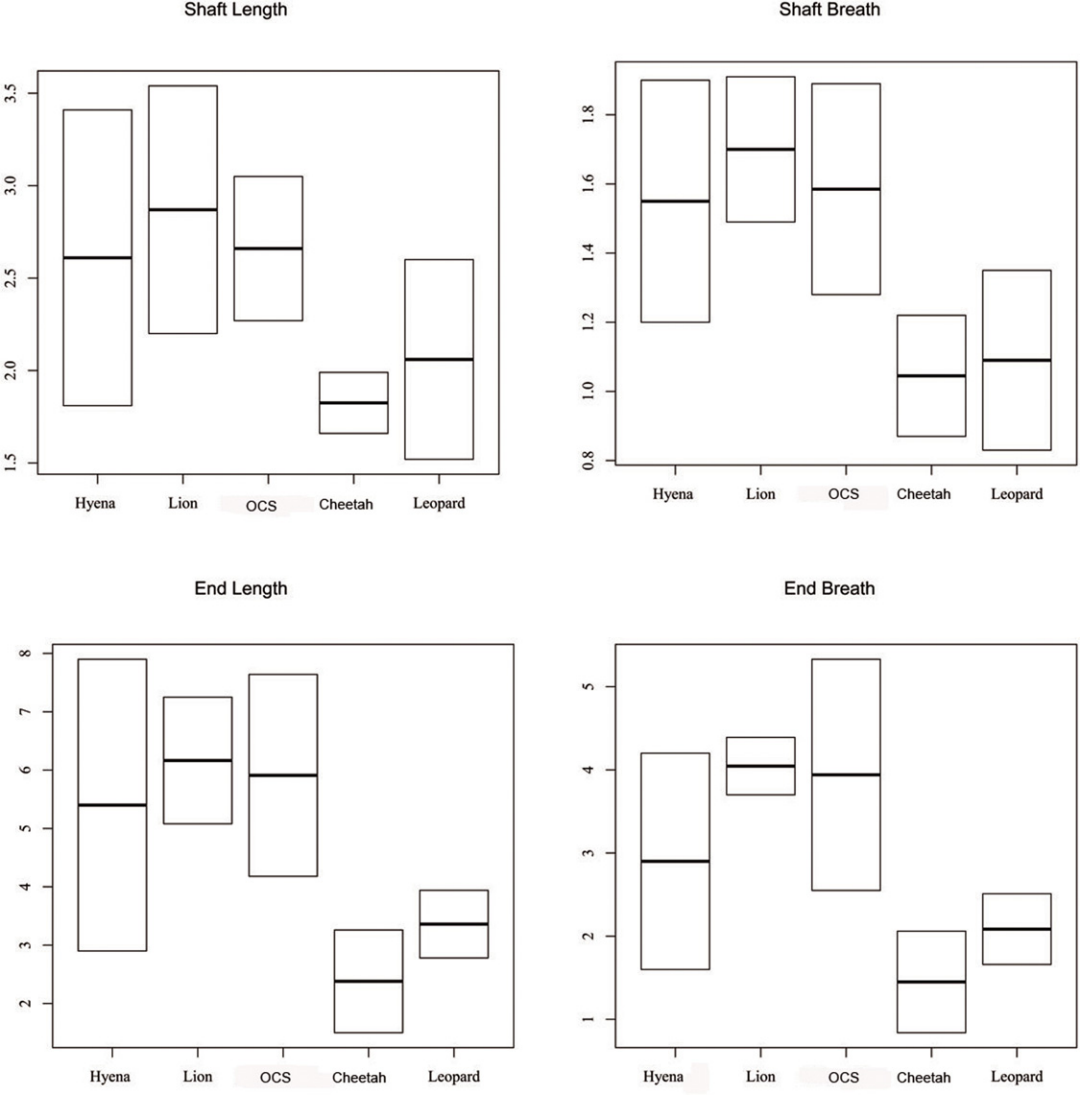
Pits size for the spotted hyena, lion, OCS, cheetah and leopard. Data for the spotted hyena and lion from ref 78. Data for the cheetah and leopard from [30].

Tooth pit size is shown in Fig 9. The size of pits on shafts shows extensive overlap between small (e.g. leopard) and large carnivores (e.g. spotted hyena and lion) and the OCS sample. Overall, the OCS sample matches the pit size recorded from large carnivores (e.g., spotted hyena and lion), but the taxon involved cannot be differentiated.

## Discussion

All the taphonomic results support a felid agency in the formation of the Olduvai bone assemblage. Given the lack of direct observation of the formation of OCS, our interpretation is inferential. Alternative hypotheses, such as a catastrophe, natural death or other carnivores present several conflictive interpretations and none of them fit the data well (see Table M in S1 File). We will discuss this in the following sections, using the main taphonomic variables analyzed. We understand that decades of research have not documented any accumulations made by lions. However, we also report here (and in [65]) that the purported lion behavior that may account for this bone accumulation is rather exceptional and caused by specific ecological factors that determine that the area is occupied by nomad lions. We do not believe that lions accumulate bones systematically as other carnivores (e.g., hyenas and, more sporadically, leopards) do. However, we believe that under certain conditions, such as those reported below, they certainly may do it.

### Skeletal part representation

Skeletal part representation is one of the diagnostic features used to discern the carnivore(s) responsible for bone accumulations recovered at modern dens and archaeological sites. Felids usually transport more complete carcasses [51,82] than the dismembered skeletons transported by hyaenids to their dens. Sometimes carcasses consumed by felids show anatomical connection and most or all the bones of the skeleton are represented [51]. In contrast, spotted hyaenas sometimes carry partial carcasses or single bones to their dens: lactating females for cubs and subadults for individual consumption, or occasionally because prey was killed close to the den [83–85]. Partial carcasses of small bovids such as gazelles are sometimes carried to dens [85], but spotted hyaenas generally accumulate isolated bones [45]. Complete wildebeest skeletons are represented at the Olduvai assemblage (Table A in S1 File). Some nearly complete wildebeest carcasses were recovered during the excavation process (Fig 1). Fig 2 shows these differences in skeletal part representation depending on the carnivore type (felid or hyenid) according to PCA. Fig 2 also shows that the OCS is most similar to the skeletal part representation documented in leopard dens. The skeletal part representation from the OCS (with some complete wildebeest carcasses) is similar to the characteristic skeletal representation documented for felids (Fig 2). The cluster analysis and RF also support this assertion (Fig 3). The probability of the OCS having been generated by felid action is higher than 75% according to RF. However, the skeletal part representation may be conditioned by other variables distinct from the differential transport of prey carried out by carnivores. The bone density from the sample of wildebeest skeletons [70] shows positive correlation with the %MAU values, which means that there is a bone density bias in the preservation of the assemblage (Fig 4). This would not be expected if lions alone had formed the assemblage. It indicates that a durophagous carnivore may have intervened. These results suggest that a felid produced the bone accumulation and a hyaenid further modified it.

Despite the completeness of the skeletons recovered, some bones are less frequently represented, such as ribs and vertebrae (Table A in S1 File). Spotted hyenas usually destroy the axial skeleton (ribs/vertebrae) during the consumption of their prey [7,22]. The low frequency of these elements at OCS (in relation to MNI) could indicate that spotted hyenas also intervened in the modification of the bone assemblage. Moreover, orientation patterns of the sample show that some physical process modified the assemblage. Maybe the water flow occurred during the wet season transported part of the bones downslope and may have modified the original skeletal part representation. Other processes such as trampling may have contributed to the documented anisotropy too.

### Breakage patterns

Of the extant large-bodied carnivores, felids are the most specialized flesh-eaters, with teeth developed almost exclusively for meat-slicing (although there are some exceptions, such as the jaguar). They primarily eat the meatier and softer parts of a carcass. Many of their physical features show adaptations toward obtaining flesh by capturing and killing animals that they then consume [5]. On the other hand, hyaena dentition is adapted for breaking bones to extract nutrients [44]. Thus, bone breakage patterns are different depending on the carnivore type (i.e. felids vs. hyenids) involved in the consumption of the prey. Felids do not fragment long bones as frequently as canids and hyaenids [37,86]. OCS preserves 209 complete long bones, which comprises about 47% of the long bone elements. The PCA performed with the number of complete long bone elements from two spotted hyaena dens (Masai Mara and Amboseli, data from [48]), clearly separates the patterns found at spotted hyaena dens from that found at OCS (Fig 5). However, 221 long bone specimens show exclusively green fracture (Table F in S1 File). The fracture angle suggests that the bone breakage was carried out by a carnivore. The angles of the oblique fractures can be attributed to the static loading characteristic of carnivore bone breakage (Table H in S1 File). When comparing the circumference section type of long bones, no correlation between the breakage patterns found at spotted hyaena dens and those recovered from the OCS is documented (Fig 6). Bone breakage carried out by spotted hyaenas generates assemblages where Type 1 is predominant [10,87]. The circumference type which predominates at the OCS is Type 3 (complete section), which means that this bone assemblage is less fragmented than those bone assemblages broken by spotted hyaenas (Fig 6). The number of notches documented at the OCS is 37, which comprises only 0.8% of the sample. Spotted hyaena dens show higher frequencies of notches, for example 16.7% of them are documented at the Masai Mara spotted hyaena den [29]. Thus, the long bone breakage pattern from the OCS surely was conducted by a carnivore, but the complete long bone frequencies, circumference type distribution and the frequency of notches show no correlation with the intensity of bone breaking inflicted by spotted hyenas in their dens.

One of the most diagnostic features of felid damage is the relatively high frequency of complete bones [37]. The breakage pattern described at the OCS does not correspond with previously documented felid behaviour. The skeletal part representation suggests a felid as the primary accumulating agent. The breakage pattern found may correspond with a secondary access by another carnivore type (i.e. hyenid) to the carcasses accumulated (see also discussion in the tooth mark section). This could explain why some bones are fragmented, which is unusual after felid consumption, and why the breakage patterns do not correspond with the damage found at spotted hyaena dens, either. Spotted hyaenas may have intermittently scavenged some bone elements from the assemblage.

### Bone surface modification

OCS shows a very low frequency of specimens bearing tooth marks. Only 7% of the sample shows some kind of carnivore damage, including furrowing on epiphyses. This is an extremely low frequency compared to the frequency found at spotted hyaena dens. For instance, two spotted hyaena dens, Syokimau and Kisima Ngeda den 2 [60,63], show a frequency of 34.8% and 44%, respectively. As we have seen above, the complete wildebeest skeletons are represented. Spotted hyenas usually create bone assemblages where limb elements predominate, because the axial skeleton (vertebrae/ribs) is consumed at kill sites [7,22]. OCS contains 363 vertebra elements (leaving out the caudal elements). Only 20.7% of these elements show carnivore damage. It has been argued that tooth marking on the ends on the apophyses may be a characteristic pattern of felid consumption of vertebrae [37,79]. The most affected elements after felid consumption are the thoracic and lumbar vertebrae [79]. OCS shows the same patterns: only 3.5% (n = 5) of cervical vertebrae are modified, while 25.5% (n = 41) of thoracic vertebrae and 50.9% (n = 29) of lumbar vertebrae were modified. After felid consumption of the vertebrae, the centrum remains intact [79]. Only 7 vertebrae from the OCS show damage on the centrum, while the remainder (n = 68) show damage only on the apophyses (Fig A in S2 File). The modification pattern of the axial skeleton from the OCS shows the same pattern that has been documented for felids. Thus, the total number of tooth-marked specimens and the carnivore damage documented on the axial skeleton do not show the pattern expected if spotted hyaenas were the primary agent in the consumption sequence of the carcasses. Conversely, these features point again to a felid as the accumulating agent of the wildebeest carcasses.

However, the access order of the carnivores to the bone assemblages has been traditionally tested through tooth marks frequencies registered on long bones. This is because most of the methodological approaches developed for carnivore taphonomy have been created to test whether carnivores or humans were the primary agents responsible for bone accumulations at sites. Blumenschine [17] showed that the frequencies of tooth marks on different portions of long bones (epiphysis, near-epiphysis or shaft), could indicate the access order of carnivores to carcasses. Primary access to carcasses by spotted hyenas shows a high percentage of midshaft fragments bearing tooth marks (>75%). Secondary access by the spotted hyena to the simulated archaeological sample created by Blumenschine shows a very low frequency of tooth-marked midshaft specimens (5–15%). According to these observations we would expect tooth mark frequencies of >75% on the midshaft fragments in the bone assemblages created by the spotted hyena. The OCS assemblage shows 15.3% of midshaft specimens bearing tooth marks. This is a very low frequency compared with the 75% expected after spotted hyena consumption of the carcasses [17–18]. Hence, the frequency of tooth marks on the midshaft again shows that the pattern documented at the OCS does not match with that expected if the spotted hyaena was the primary agent accumulating the bone assemblage.

Actualistic studies have also been conducted with carcasses consumed by lions in the wild [79–88]. These studies show that the humeri and the femora are the most frequently tooth-marked bones and the tibia the least tooth-marked element. The OCS assemblage shows the same carnivore damage frequencies on the long bone elements (Table K in S1 File). On the other hand, almost 99% of all complete long bones from the carcasses consumed by lions bore less than ten marks on the shaft [88]. This feature was also found with carcasses consumed by leopards: complete long bones rarely show more than 3–5 tooth marks [86]. Therefore, the low frequency of tooth marks on midshaft of complete long bones is characteristic of primary access of felids to carcasses. All the long bones from the OCS bear less than 10 tooth marks on the shafts. The low frequency of tooth marks on the shafts indicates that a felid consumed the meat of the wildebeest. Moreover, lions inflict fewer tooth marks on the shafts (6.1%) than on the epiphysis (35.1%) [88]. However, the frequency of tooth-marked specimens on epiphyses (5.6%) is lower than on shaft specimens (15.3%) at the OCS (Table K in S1 File). As we have seen earlier, the spotted hyena may have acted in the modification of the bone assemblage by scavenging part of the elements, given the breakage patterns and the skeletal part representation results. Surprisingly, the percentage of midshafts bearing tooth marks from the OCS (15.3%) is consistent with the expected percentage (5–15%) in secondary access of spotted hyenas to an archaeological site [17]. Bone-crushing carnivores can significantly bias the skeletal part abundances by deleting less-dense limb bone epyphyses [19,21]. The variables which quantified the bone destruction inflicted by the different carnivores (furrowing patterns and taphotypes) may shed more light on this discussion. Fig 7 shows that there are differences between the furrowing patterns recorded at the spotted hyena dens and those documented at lion kills. Nevertheless, the furrowing pattern from OCS does not match any of the two carnivores compared. Likewise, Fig 8 shows that the taphotypes reported from the spotted hyena and lion kills are different. Again, OCS shows no direct correlation with the taphotypes of these two African carnivores. But, when the confidence intervals are introduced (Fig 8), we can see that distinctive lion and spotted hyena taphotypes are included in the OCS sample. Accordingly, we can conclude that both carnivores inflicted furrowing damage on the wildebeest long bones. The secondary access of the spotted hyena to the assemblage may delete some long bones epiphyses, thereby producing a decrease in the number of epiphyses bearing tooth marks.

On the other hand, it has been argued that pits size could be used to distinguish small from large carnivores [64,78,89–91]. Pits > 4 mm. have been observed in carcasses consumed by large carnivores (e.g., hyenas, lions, large dogs), but smaller tooth marks could be attributed to both small and large carnivores [64,78,90–91]. Fig 9 shows some overlapping in the size of pits on shafts from the OCS and those inflicted by the leopard, lion and spotted hyena. Overall, the result from OCS matches those expected after the consumption of prey carried out by a large carnivore (e.g. lion, spotted hyena), but the taxon involved cannot be discerned through this analysis.

In sum, the number of tooth-marked specimens, the carnivore damage documented on the axial skeleton, the frequency of tooth-marked midshafts and the number of marks on shafts per complete long bone suggests that felids were the prime consumers of the carcasses. The frequency of tooth-marked epiphyses and midshafts, furrowing patterns and taphotypes show that both lions and spotted hyaenas modified the bone assemblage. Given that lions do not usually follow hyenas in bone modification, it is safe to assume that lions preceeded hyenas in bone modification at the Olduvai assemblage. The presence of complete long bones and elements of the axial skeleton also indicate that hyenas only partially modified the bone assemblage.

### Carnivore behavioral ecology

Lansing et al. [85] pointed out the importance of combining taphonomic approaches with carnivore behavioural ecology. One of the features of predator behaviour studied at hyena dens is the number of items collected and transported by these carnivores. However, little data are currently available on accumulations rates in relation to den occupation patterns. The accumulations at spotted hyena dens tend to form more slowly than those of other hyenas species, likely due to their gregarious behaviour and the high intraspecific competition for food [85]. Some estimations of the number of bones collected by spotted hyenas, the only hyena species present in the Serengeti ecosystem, show a rate of 1.3–9 specimens per month [72,84,85,92]. OCS shows an accumulation rate of 426 specimens per year. The wildebeest migration reaches the Serengeti plains (where Olduvai Gorge is located) around November or December and move eastward into the Serengeti Corridor once the plains dry out in May or June, when the dry season begins [93]. An 8-month span was used to calculate the accumulation rate: 53 items per month. This shows a higher number of specimens collected than those reported for the spotted hyena and may be even higher since the bone accumulation seemed to be produced only during the early wet season (from November to January) [65]. Again, this feature is not consistent with the expected behaviour of the spotted hyena.

Carnivorous mammals bigger than 20 kg in body mass usually hunt prey equal to or larger than their own body size [94]. The large carnivores present in the Serengeti ecosystem are: the spotted hyena; African wild dog (although African wild dogs became extinct in the Serengeti-Mara ecosystem in 1991, in 2001, wild dogs naturally re-established in this area [95]); cheetah; leopard; and lion, all of them exceeding 20 kg in body mass [1]. The leopard, African wild dog and cheetah show a prey range with lower body mass than those of the blue wildebeest [1]. Furthermore, the blue wildebeest (the only species registered at OCS) is significantly avoided by these three African predators even though they co/occur in the same ecosystems [96–98]. Of these three carnivores, the only one which has been reported as an accumulating agent in the savannah ecosystem is the leopard [8]. Kruuk and Turner [99] showed that the leopard preyed on only one adult wildebeest in their observations carried out in the Serengeti plains during eight years. The leopard from the Kruger National Park (South Africa) only preyed upon two adult wildebeest in a period of two years [100]. Furthermore, the leopard diet is more varied than that of the lion [1]. In fact, the two main predators of wildebeest in the Serengeti are the spotted hyena and lion [101]. Analysis of sex and age classes of the wildebeest recovered were carried out and compared with data of spotted hyena and lion kills in order to ascertain the carnivore responsible for the accumulation [65]. The results showed that the age selection by these predators in the Serengeti depend on the growth rate of the ungulate population, seasonality, and/or habitat. There are no data available with all these criteria for the Serengeti ecosystem, and the five-age class method is the only one able to distinguish between the diet of the spotted hyena and lion [65]. The wildebeest bone accumulation was produced in the early wet season, when both the lion and spotted hyena select an equal sex ratio in their kills [65]. Thus, age and sex class selection are not valuable data to discern the carnivore responsible for the OCS bone accumulation, but these analyses (using the five-age class method) may have great potential for discerning anthropic and paleontological sites of the Pleistocene, as in the case of Olduvai archaeopaleontological sites. As we mentioned earlier, the age-class profile does not correspond with a catastrophic event [65]. Previous to this finding, some modern bone accumulations have been documented in an escarpment of Olduvai Gorge, representing a wildebeest herd with broken legs in tree branches [102]. The interpretation for this bone assemblage was that a cloudburst had flooded the plains and swept a whole herd over the edge [102].

Although both predators (spotted hyena and lion) hunt wildebeest in the Serengeti, there are some differences in their hunting behaviour. While the lion prey preferences are significantly predicted by the body mass of their prey [103], the same analysis revealed that there were no factors that significantly predicted the Jacobs´ index value of spotted hyaena prey [104]. The Jacobs´ index assess the proportion of kills made by a carnivore along with the proportional abundance of that species in the ecosystem [96]. The range of weight of the prey species preferred by the lion is 190 to 550 kg, irrespective of their availability [103]. In the Serengeti, lion preferred prey ranging from 170 to 250 kg [105]. When all habitat types (woodlands, open plains, etc) are analyzed, the lion significantly prefers five prey species (blue wildebeest, gemsbok, buffalo, giraffe and plain zebra) and avoid 11 other prey species [103], which means that the hunting behavior of the lion is very specialized. The dependence of medium-size ungulate species, such as the blue wildebeest and zebra, is still higher in the open plains [99], the habitat type of Olduvai Gorge in the Serengeti ecosystem. In contrast, the spotted hyena diet is more flexible [100], and they do not exhibit a significant preference for any species and avoid very few [104]. Bone assemblages created by spotted hyenas reflect species availability and local environment [106]. From one research season in 2012 to the next in 2013, the carnivore accumulated 6 new individuals. This implies that OCS has been active for several years (NMI = 55). No bones belonging to species other than the blue wildebeest were found (NISP = 4533). It is not likely that spotted hyenas were the agents that accumulated the bones at OCS. The spotted hyena does not exhibit any preferences for specific prey and it is a generalist predator. It, thus, rarely creates a bone assemblage with only one ungulate species over several years. The Serengeti ecosystem shows more than 20 ungulate species and some of them occur at high population densities in the grassland unit, such as the zebra or Thompson´s gazelle [1,67]. OCS is a modern site made by carnivores, containing the highest number of mammal specimens documented, and the only one with just one prey species in all of the carnivore assemblages known in Africa. Although the number of specimens is not a constant variable at hyena dens [57] it seems that the number is rarely higher than 1000 bones [62]. In general, the assemblages of striped and brown hyenas (both species missing in the Serengeti ecosystem) seem to be larger than those created by the spotted hyena [59,62,85]. All three hyena (spotted, striped and brown) and modern leopard dens show different kinds of species and animals of a wide body mass [8,48,51,59,62,85]. Since the OCS assemblage shows the largest collection of mammal bone specimens and it is unique in terms of having only one ungulate species, the carnivore which created the assemblage may be a specialized predator, and the blue wildebeest may be one of their preferred prey. The only carnivore present in the Serengeti ecosystem which exhibits these features is the lion.

In addition to population densities and hunting behaviour, predator-prey dynamics are contingent on the peculiarities of specific ecosystems [107]. These authors have shown that lions in the plains select areas where prey is easier to catch, rather than areas where prey densities are higher. The chance of a kill or scavenged carcass by lions on the plains increased with proximity to water and the degree of terracing [107]. The topography of the OCS enables an ambush predator to stalk, and proximity to water allows the possibility of encountering prey. Since the ease of capturing prey should be considered essential to models of habitat selection by an ambush predator (such as the lion) as opposed to cursorial predators [107], the habitat type of the site may suggest ambush instead of cursorial predation. In fact, spotted hyenas (cursorial predators) prefer open habitats over the relatively dense woodland that flanks perennial rivers [45], such as the environment located close to OCS. The most vulnerable place for lion prey is in the vicinity of riverine thickets, not only because of the denser vegetation there, but because prey is restricted in terms of escape routes [1]. Most lion prides move eastward during the rains to reach the prey concentrations on the intermediate grasslands [1]. Nomad lions are the most likely kind of lion to reach short-grasslands (the Serengeti ecological unit where OCS is located), where they sometimes establish temporary territories in the plains, or settle down in one locality for months, going to the woodlands for the dry season, but returning to the same area during the following rains [1,108]. Lions with little or no cover, as in plains habitats, catch fewer animals [1], it is natural that small groups of nomad lions (on average 2.8 individuals) in the short grassland ecological unit select the vicinity of riverine thickets for hunting. Although it may be difficult to imagine a lion dragging a prey to a location away from the place where it was caught, Schaller (ref [1]; pages 267–268) reported how the lion can carry adult wildebeest and zebra kills up to 160 m: “Large kills are transported in two ways, as shown by a male which had just killed a zebra. First, he backed up, tugging violently at the throat of his kill, and moved the body 20 m in this fashion. Then he bit it in the nape, and, with the neck of the carcass between his forelegs, pulled into 23 m into a ravine, taking a succession of rapid steps before pausing briefly”. A tooth-mark was found on an atlas vertebra (Fig G in S2 File), which may correspond to this behavior described in lions. The regular visits of lions to this spot in the gorge may generate the accumulations of the carcasses consumed by the group. The topography of the site, although it is not a cave or a modified burrow, propitiated the bone accumulations through years, because despite some carcass transport being produced by heavy rains in the wet season, some of the bones were buried in the site´s slope. As we have noted earlier, other kinds of carnivores, such as the spotted hyena may have acted in the modification of the bone assemblage, too. Bone breakage patterns, furrowing patterns and taphotypes show that a bone-crushing carnivore consumed some of the bones. However, the intensity of damage generated by the spotted hyena in the bone assemblage is not very high because they prefer open vegetation habitats [45], not the relatively closed habitat of the site.

Sociality, philopatry and dispersal decisions in lions have been the aim of research during the last decades [2,109–111]. Female lions show philopatry, but up to one-third of subadults females disperse [112]. When a new coalition of males takes over a pride, it evicts the previous resident males and the male and female subadults [110,113–114]. The resources which limit population sizes in species regarded as philopatric are food and den sites [115]. Dispersing female lions show lower reproductive success [109,110,112] but they may know new safe denning sites and good hunting areas [109]. Most solitary females quickly acquire mates [110]. Recently, Craft et al. [116] pointed out that although migration has been proposed as temporary refuge to escape predation for herbivores [117], large territorial predators such as hyenas, jackals and lions can adapt to the movements of the migratory ungulates. Thus, it is possible that ungulates deal partially with predation [65,116]. Although lions do not breed seasonally [1, 113,114,118], nutrition affects reproductive condition [1,113,119]. We hypothesize that the southernmost areas of the Serengeti ecosystem may present suitable denning sites close to seasonal rivers with available migratory prey and it may benefit lion reproduction. Hanby and Bygott [110] emphasized the importance of long-term demographic and ethological studies in the different habitats which comprise the Serengeti ecosystem. Further research is needed in order to confirm the hypothesis presented here and to show the behavioral differences (whether they exist) between nomadic individuals and lions in prides.

## Conclusions

The study of the skeletal part representation, the low breakage on long bones detailed by the higher frequency of circumference type 3 and low frequency of notches, the low frequency of tooth marks and their distribution on long bones, and the modification pattern of the axial skeleton (including the tooth mark registered on an atlas vertebra), suggest that a felid was the most likely accumulating agent at OCS. Moreover, the low frequency of the axial skeleton, tooth pit size ranges, the furrowing patterns and the taphotypes documented indicate that both the lion and spotted hyaena modified the bone assemblage. The accumulation rate, body mass of the prey, the specialized-pattern of only one ungulate species, and the topography and environment of the site suggest that the primary accumulating agent was a felid (probably a lion), although the spotted hyena scavenged part of the bones. This bone assemblage potentially created by lions constitutes a new framework for taphonomic studies. The use of these data in early Pleistocene sites may strengthen the hypothesis regarding felid accumulation in the FLK N site at Olduvai Gorge (Tanzania) as suggested by previous taphonomic work [29]. Furthermore, the data presented here may also require rethinking of the interpretation of important classical sites for human evolution. The new data presented also may be useful in future taphonomic research carried out in archaeological and paleontological sites from Africa, Europe, Asia, North America and the northern part of South America, where large lion-like felids were present in the paleoecosystems during the Pleistocene [5,120]. This study also draws attention to the claims against non-anthropogenic authorship of assemblages where only one taxon is the documented prey. Recently, the hominin accumulations documented at the 400 ka site of Sima de los Huesos (Atapuerca, Spain) or in the Dinaledi Cave (Rising Star, South Africa), with unknown age, have been argued to have an anthropogenic origin, since no known carnivore has been documented to prey on just one species. The present study shows that single-taxon prey assemblages are produced (at least, marginally) by some modern carnivores.

## Supporting Information

S1 File Tables. (DOCX)

S2 File Images. (PDF)
