# Supplementary material for: Lions as Bone Accumulators? Paleontological and Ecological Implications of a Modern Bone Assemblage from Olduvai Gorge
Source: PLoS One. 2016 May 4;11(5):e0153797. doi: 10.1371/journal.pone.0153797 (PMC4856334; doi:10.1371/journal.pone.0153797)
Supplement: S1 File — (DOCX) [file pone.0153797.s001.docx]

**Supporting Information**

S1: Tables.

Table S1A. Skeletal part representation data from OCS.

| Element | NISP | MNE | NISP/MNE | MAU | % MAU |
| --- | --- | --- | --- | --- | --- |
| Maxilla/Skull | 194 | 23 | 8.4 | 23 | 49.5 |
| Hemimandible | 223 | 63 | 3.5 | 31.5 | 67.7 |
| Cervical | 159 | 145 | 1.1 | 20.7 | 44.5 |
| Thoracic | 262 | 161 | 1.6 | 11.5 | 24.7 |
| Lumbar | 113 | 57 | 2 | 9.5 | 20.4 |
| Caudal | 88 | 84 | 1.1 | 5.3 | 11.4 |
| Ribs | 1622 | 349 | 4.7 | 15.9 | 34.2 |
| Scapula | 119 | 93 | 1.3 | 46.5 | 100 |
| Innominate | 98 | 38 | 2.6 | 38 | 81.7 |
| Humerus | 112 | 64 | 1.8 | 32 | 68.8 |
| Radius | 104 | 54 | 2.08 | 27 | 58.1 |
| Ulna | 55 | 48 | 1.2 | 24 | 51.6 |
| Metacarpals | 154 | 87 | 1.8 | 43.5 | 93.6 |
| Femur | 86 | 41 | 2.1 | 20.5 | 44.1 |
| Tibia | 145 | 76 | 1.9 | 38 | 81.7 |
| Metatarsals | 119 | 79 | 1.5 | 39.5 | 85.0 |
| Carpals/Tarsals | 256 | 245 | 1.05 | 11.1 | 23.9 |
| Phalanges | 317 | 302 | 1.05 | 12.6 | 27.1 |
| Others (sacrum+sternum+  sesamoid+patella+malleolus) | 127 | 61 | 2.08 | 2.03 | 4.4 |
| Metapodium | 10 | 0 |  |  |  |
| Vertebrae | 170 | 0 |  |  |  |
| TOTAL | 4533 |  |  |  |  |

Table S1B. Correlation values and their significance for each of the variables and the associated component for the skeletal part representation (%MAU), according to PCA.

| **Dimension 1** | **correlation** | **p-value** |
| --- | --- | --- |
| Rib | 0.979 | 0.0001 |
| Innominate | 0.967 | 0.0004 |
| Ulna | 0.951 | 0.0010 |
| Cervical vertebrae | 0.944 | 0.0014 |
| Metacarpals | 0.929 | 0.0025 |
| Thoracic vertebrae | 0.923 | 0.0031 |
| Hemimandible | 0.909 | 0.0045 |
| Carpals/Tarsals | 0.892 | 0.0070 |
| Radius | 0.856 | 0.0140 |
| Metatarsals | 0.850 | 0.0154 |
| Femur | 0.849 | 0.0158 |
| Scapula | 0.826 | 0.0221 |
| Humerus | 0.816 | 0.0253 |
| **Dimension 2** | **correlation** | **p-value** |
| Lumbar vertebrae | 0.785 | 0.0367 |
| Tibia | 0.773 | 0.0417 |
| Maxilla/Skull | -0.900 | 0.0057 |

Table S1C: Statistical tests applied to the orientations from the OCS sample and their significance.

|  | Test | p |
| --- | --- | --- |
| Rayleigh´s test | 0.57 | 9.8 x 10^-35^ |
| Kuiper´s test | 6.30 | <0.01 |
| Watson´s test | 4.06 | <0.01 |

Table S1D: Number of complete limb bones recovered from the OCS.

| Element | Number of Complete Bones (n=209) | % per NME |
| --- | --- | --- |
| Femur | 21 | 51.2 |
| Humerus | 28 | 43.75 |
| Metacarpus | 53 | 60.9 |
| Metatarsus | 50 | 63.3 |
| Radio | 22 | 40.7 |
| Tibia | 34 | 44.7 |
| Ulna | 1 | 2.08 |
| TOTAL | 209 | 46.6 |

Table S1E. Correlation values and their significance for each of the variables and the associated component for the complete long bones, according to PCA.

| **Dimension 1** | **correlation** | **p-value** |
| --- | --- | --- |
| Tibia | 0.992 | 0.0078 |
| Humerus | 0.980 | 0.0202 |
| Femur | 0.978 | 0.0224 |
| **Dimension 2** | **correlation** | **p-value** |
| Metacarpus | 0.970 | 0.0296 |

Table S1F: Fracture type according to the bone element.

| Element | Dry Breakage | Green Breakage | Dry and Green Breakage |
| --- | --- | --- | --- |
| Compact Bones | 3 | 3 | 0 |
| Long Bones | 231 | 221 | 120 |
| Axial Bones | 614 | 221 | 27 |

Table S1G: Number and type of notches identified in the OCS bone assemblage.

| Element | Number of Notches | Single (n=8, 21.6%) | B (n=10, 27.0%) | C (n=7, 18.9%) | D (n=12, 34.4%) | % per MNE |
| --- | --- | --- | --- | --- | --- | --- |
| Femur | 6 | 1 | 3 | 1 | 1 | 14.6 |
| Humerus | 18 | 4 | 4 | 2 | 8 | 28.1 |
| Radio | 4 | 0 | 2 | 1 | 1 | 7.4 |
| Tibia | 6 | 1 | 1 | 3 | 1 | 7.9 |
| Ulna | 1 | 0 | 0 | 0 | 1 | 2.1 |
| Metacarpus | 2 | 2 | 0 | 0 | 0 | 2.3 |

Table S1H: Fracture angle. Oblique fracture, size class 3. Dynamic and static values from ref 74.

|  | Dynamic | | Static | | OCS | |
| --- | --- | --- | --- | --- | --- | --- |
| Angle | <90º | >90º | <90º | >90º | <90º | >90º |
| Mean | 59.2 | 126.5 | 82.3 | 107.4 | 80.7 | 108.04 |
| S.D. | 18.1 | 15.96 | 5.1 | 8.83 | 6.8 | 11.4 |
| 95% C.I. | 55.8-62.6 | 121.2-131.8 | 76.6-88.9 | 101.4-113.4 | 75.5-85.9 | 103.1-113 |
| n | 48 | 38 | 9 | 12 | 9 | 23 |
| Range | 16-86 | 95-165 | 76-89 | 95-125 | 70-89 | 91-138 |

Table S1I: NISP with tooth mark according to element type (Furrowing not included).

| **Element** | **NISP** | **% NISP** |
| --- | --- | --- |
| Maxilla | 0 | 0 |
| Mandible | 0 | 0 |
| Cervical | 4 | 2.5 |
| Thoracic | 10 | 3.8 |
| Lumbar | 4 | 3.5 |
| Caudal | 0 | 0 |
| Ribs | 35 | 2.2 |
| Scapula | 11 | 9.2 |
| Innominate | 5 | 5.1 |
| Humerus | 25 | 22.3 |
| Radius | 23 | 22.1 |
| Ulna | 11 | 20 |
| Metacarpals | 17 | 11.0 |
| Femur | 21 | 24.4 |
| Tibia | 22 | 15.2 |
| Metatarsals | 3 | 2.5 |
| Carpals/Tarsals | 5 | 2.0 |
| Phalanges | 2 | 0.6 |
| Others(sacrum+sternum+sesamoid+patella) | 6 | 4.7 |
| **TOTAL** | 204 |  |

Table S1J: Frequency of carnivore damage on vertebrae from the OCS.

|  | Cervical | Thoracic | Lumbar |
| --- | --- | --- | --- |
| Damage on the centrum | 1 (0.7) | 1 (0.6) | 5 (8.8) |
| Damage on apophyses | 4 (2.8) | 40 (24.9) | 24 (42.1) |

Table S1K: Frequency of tooth marked specimens per element type and bone section.

| Element | Proximal | Shaft | Distal | Total with damage |
| --- | --- | --- | --- | --- |
| Humerus | 5 (4.5) | 20 (17.9) | 6 (5.4) | 31 (27.7) |
| Radius-ulna | 9 (5.7) | 22 (13.8) | 0 (0) | 31 (19.5) |
| Femur | 4 (4.7) | 15 (17.4) | 3 (3.5) | 22 (25.6) |
| Tibia | 0 (0) | 20 (13.8) | 1 (0.7) | 21 (14.5) |

Table S1L. Correlation values and their significance for each of the variables and the associated component for the furrowing patterns, according to PCA.

| **Dimension 1** | **correlation** | **p-value** |
| --- | --- | --- |
| Distal femur | 0.995 | 0.0046 |
| Proximal tibia | 0.973 | 0.0275 |
| Proximal radius | 0.955 | 0.0449 |
| **Dimension 2** | **correlation** | **p-value** |
| Proximal femur | 0.971 | 0.0193 |
| Distal humerus | 0.970 | 0.0283 |

Table S1M. Alternative hypotheses and the refuting arguments.

| Hypotheses | Refuting Arguments |
| --- | --- |
| Catastrophe | 1. The age profile of the Olduvai bone assemblage (dominated by prime adults) contrasts with the age profile of the wildebeest populations in the Serengeti (ref 65). A catastrophe, such as falling from a cliff would not sample selectively for ages, since it would be random. With Sinclair´s data (ref 121), there is a remarkable absence of old and very old individuals in the Olduvai sample. Compared to Watson´s (ref 122) data, there is a striking underrepresentation of calves and yearlings in the Olduvai sample. Given the lack of age profiling in modern wildebeest populations, the Olduvai samples presents a specialization in non-old adults and does not sample all the age classes as represented in a modern wildebeest population.  2. Likewise, a catastrophe would yield resources for scavengers, such as hyenas, which are more abundant in the area than any other medium-sized or large carnivore. The bone damage patterns documented should therefore be those of hyenas, instead of the felid modifications reported (see text descriptions and data).  3. A catastrophe specifically linked to the locus where the assemblage was formed would be explainable by a one-event process. The redundancy in the accumulation by several events (the last ones controlled by our team) argues against a catastrophic event.  4. Several of the complete carcasses were collected at the top of the slope, at the rear of the main cliff platform, where no catastrophe caused by falling could be feasible.  5. The cliff by the slope where the accumulation was formed is detached from the rest of the cliff platform by a small valley acting as a passage. Wildebeest would have needed to cross it to access the cliff, which is devoid of grass most of the year, since it is covered by bushy vegetation, non-adequate to wildebeest feeding.  6. Not a single wildebeest bone was found when surveying the cliff platform. |
| Hyena den | 1. No other taxa present. This highly specialized taxonomic accumulation has never been documented in a hyena den, which tend to be taxonomically eclectic.  2. Skeletal part profiles show the presence of complete carcasses, where axial elements are very abundant. This has also never been reported for hyena dens.  3. Bone completeness argues against hyenas as the main bone accumulators, since most bones found at spotted hyena dens are broken and cylinders (missing in the Olduvai asemblage) are abundant.  4. Bone damage patterns (anatomical distribution of furrowing and tooth marking) are very different from those reported in spotted hyena dens (see text).  5. Bone damage patterns in the Olduvai assemblage are also different from those observed during carcass consumption at kill site by spotted hyenas (MDR, personal observation). These are very similar in bone breakage and furrowing patterns to those reported for bones at hyena dens, but different in the intensity of tooth-marking. |
| Leopard bone accumulation | 1. Adult wildebeests are not part of the regular repertoire of leopard prey (ref 1 and 99)  2. Leopards are ambush predators and this prevents them from being specialists in prime adult medium-sized prey.  3. The tooth mark punctures found on several axial elements are larger than those reported for leopards (ref 30). |
| Human-made bone accumulation | 1. Not a single evidence of cut marks, percussion marks or bone breakage patterns attributed to human processing of carcasses were found. |
| Accumulation made by carnivores other than hyenas or leopards | 1. No other carnivores are known to accumulate bones in modern African savannas. |
