# Supplementary material for: Lions as Bone Accumulators? Paleontological and Ecological Implications of a Modern Bone Assemblage from Olduvai Gorge
Source: PLoS One. 2016 May 4;11(5):e0153797. doi: 10.1371/journal.pone.0153797 (PMC4856334; doi:10.1371/journal.pone.0153797)

Supporting Information

S2: Figures.

Figure A: Carnivore damage on vertebrae from the OCS.

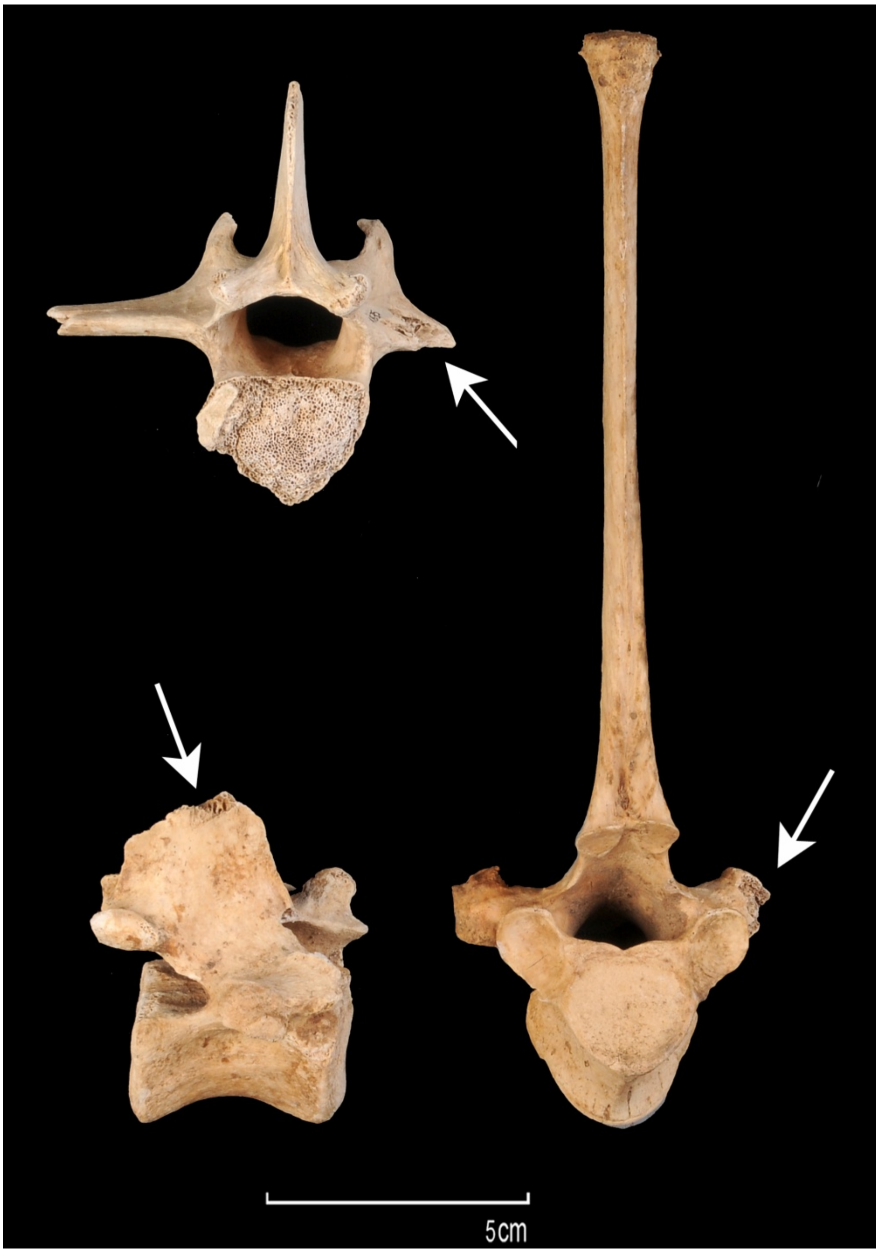

Figure B: Taphotypes for ulnae from the OCS.

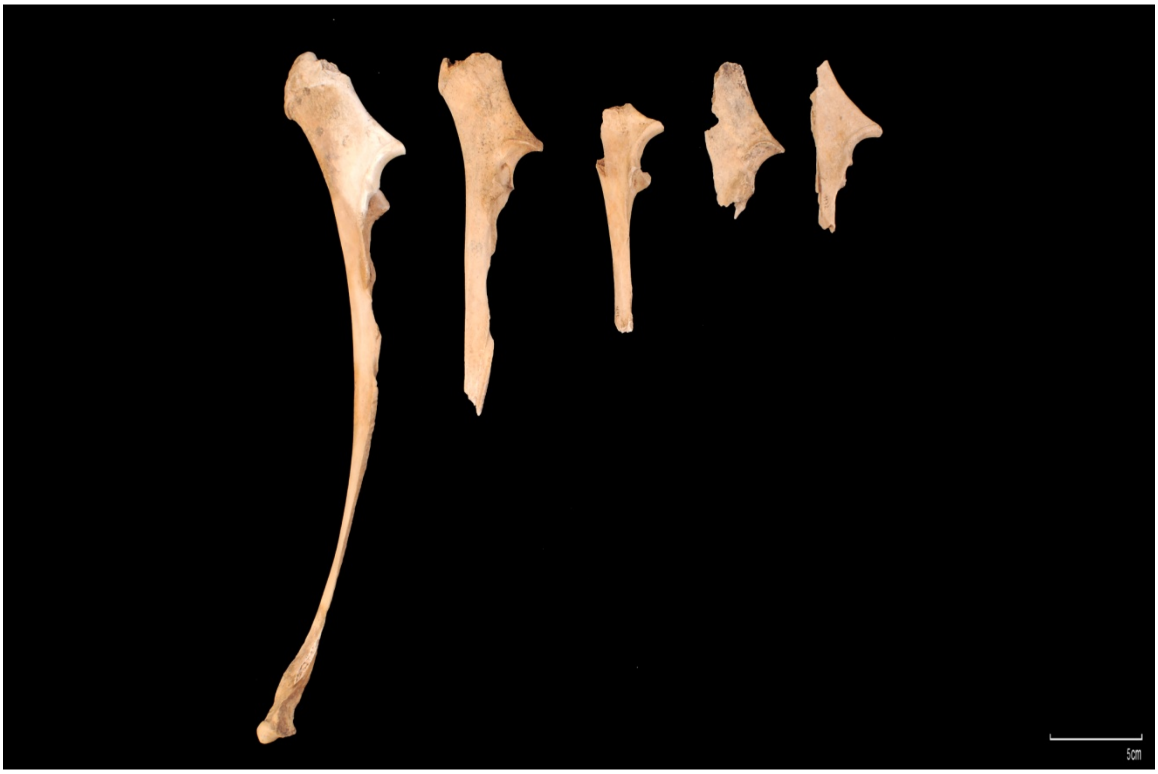

Figure C: Taphotypes for radius from the OCS.

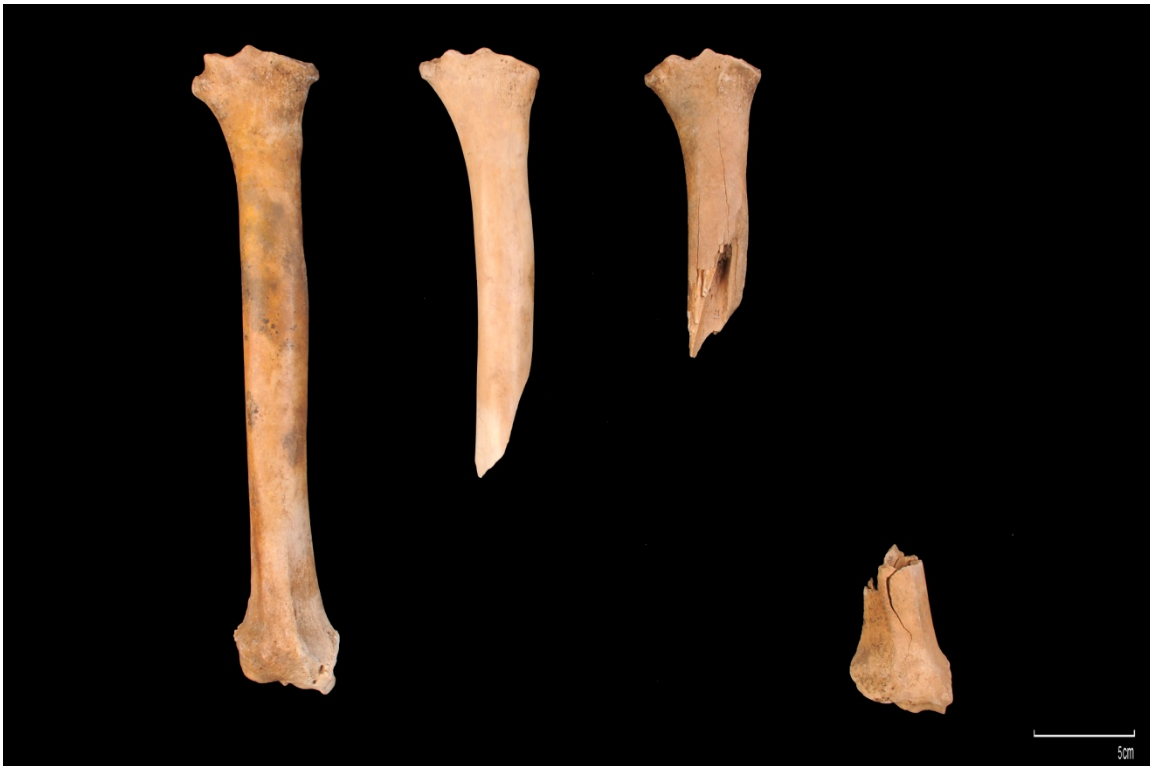

Figure D: Taphotypes for tibiae from the OCS.

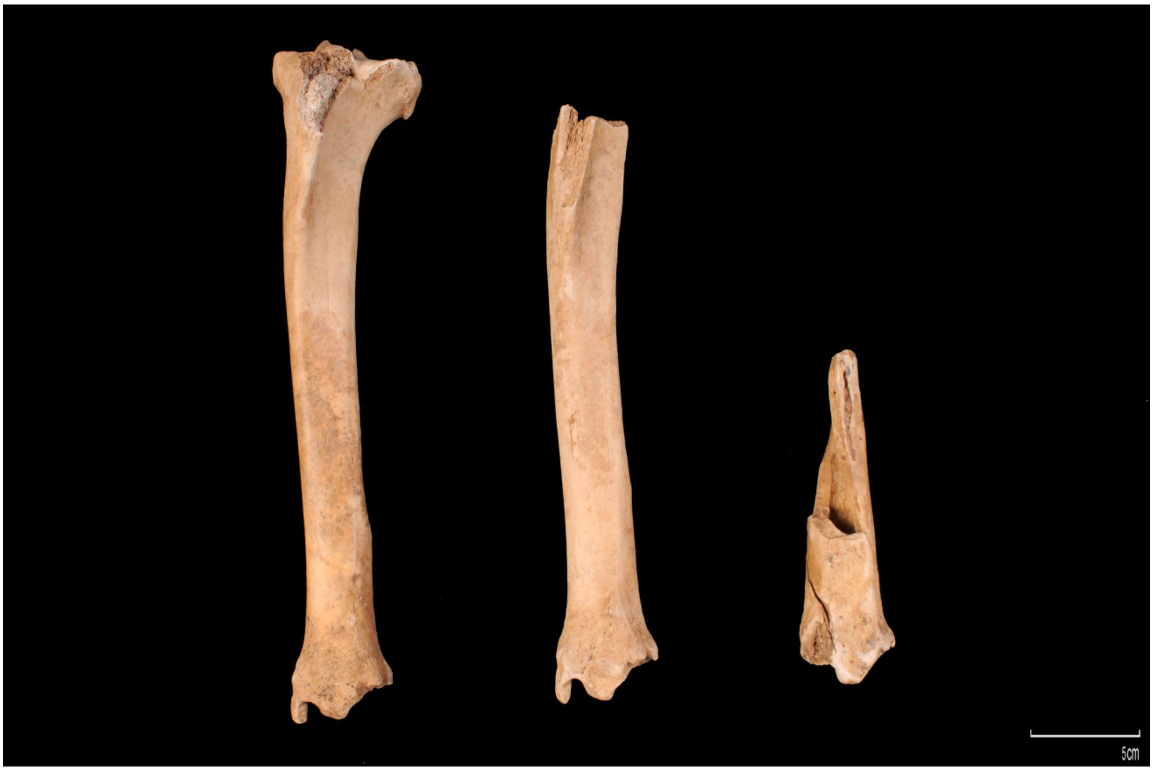

Figure E: Taphotypes for humerus from the OCS.

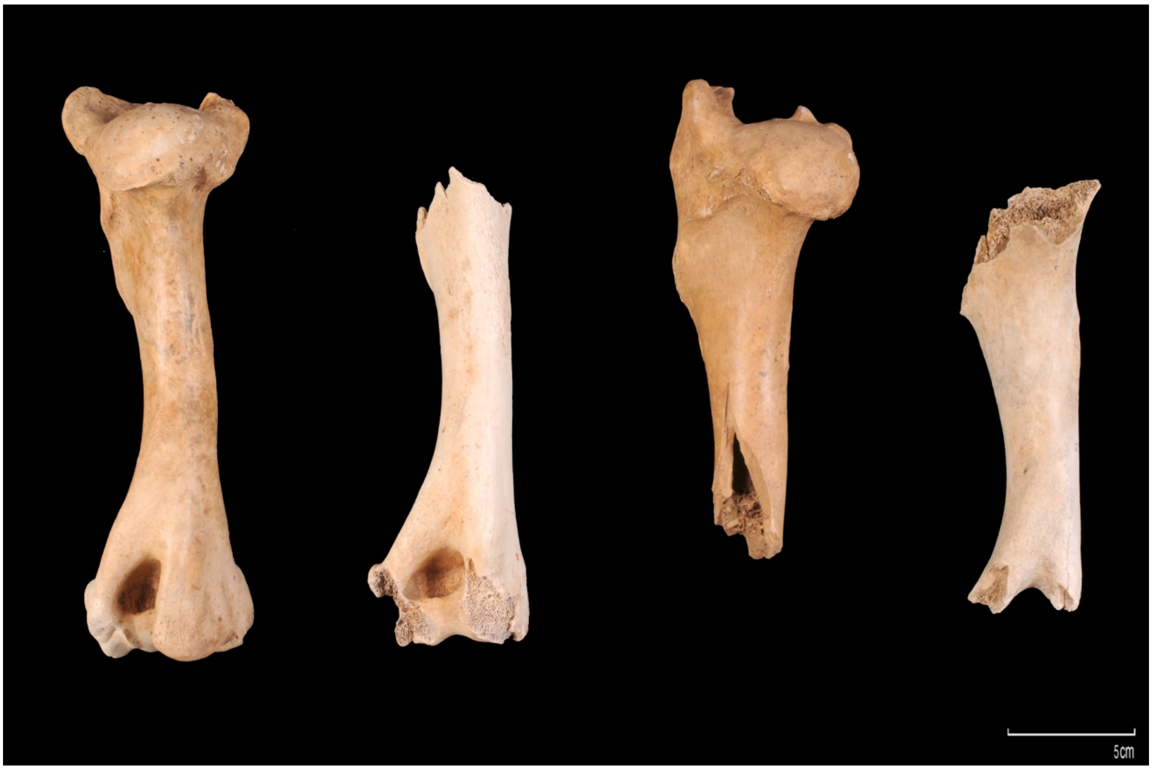

Figure F: Taphotypes for femur from the OCS.

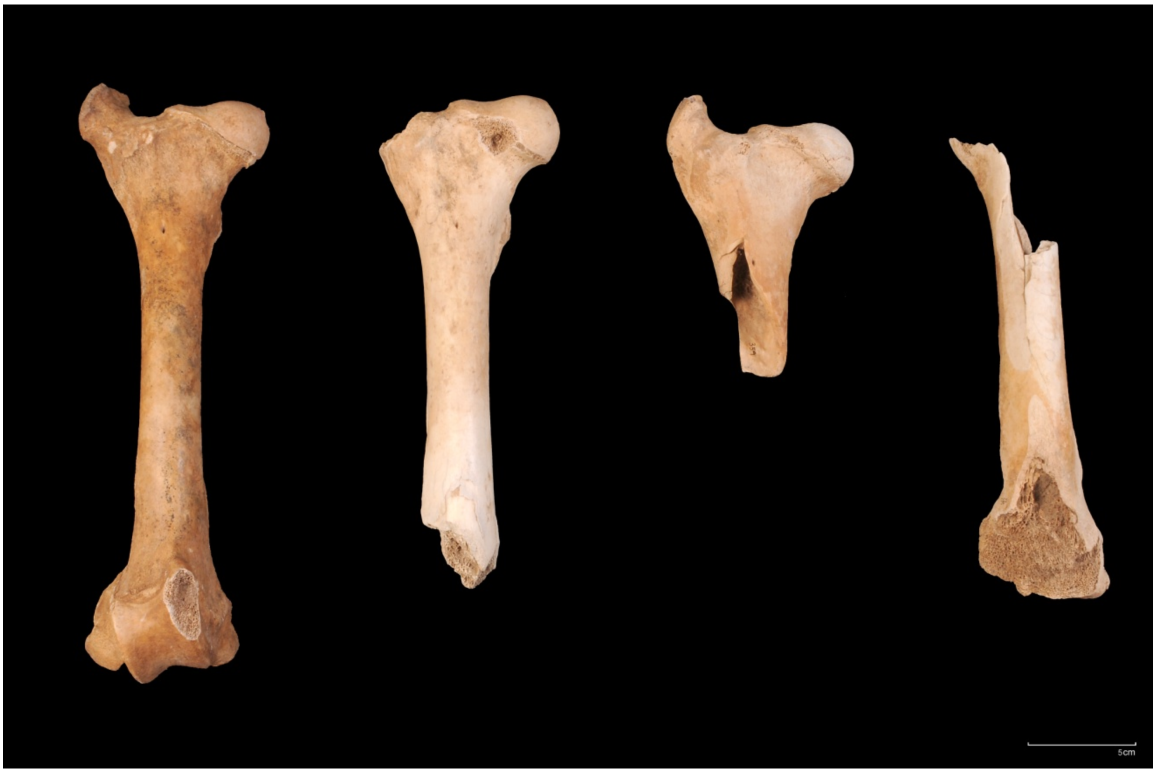

Figure G: Tooth mark on an atlas vertebra from the OCS.

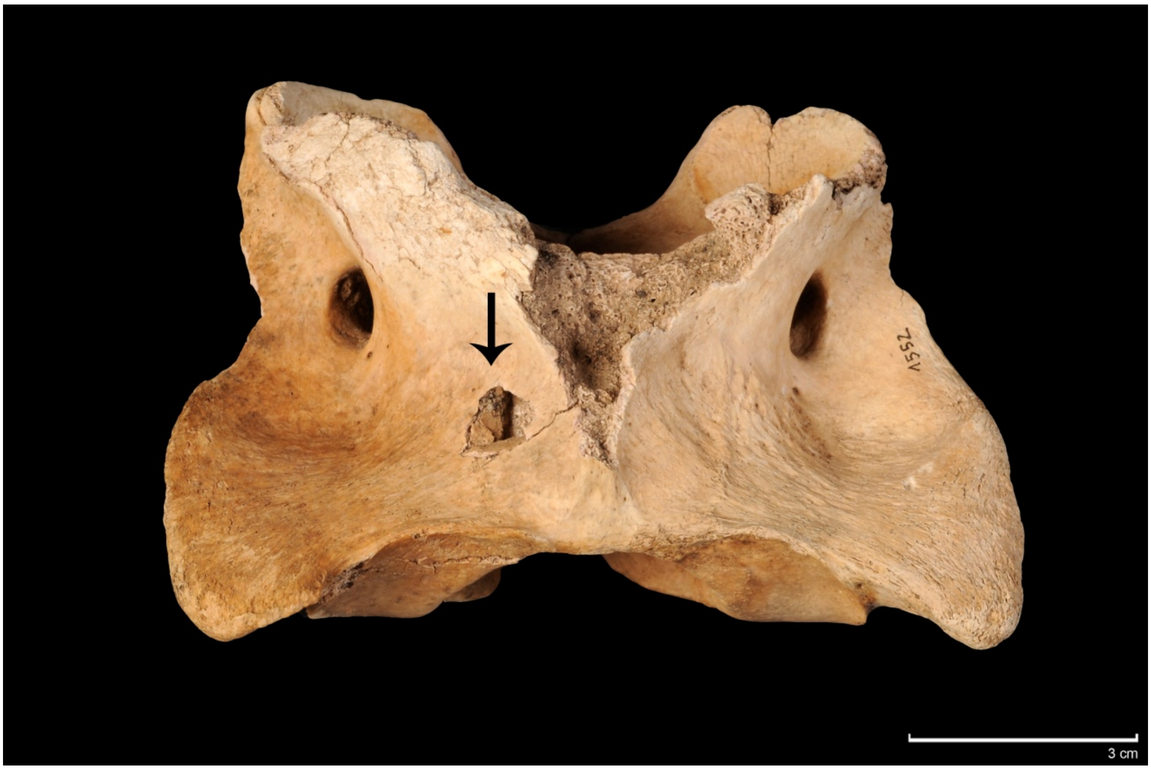

Supplement: S2 File — (PDF) [file pone.0153797.s002.pdf]
